# DC Shifts-fMRI: A Supplement to Event-Related fMRI

**DOI:** 10.3389/fncom.2019.00037

**Published:** 2019-06-12

**Authors:** Qiang Li, Guangyuan Liu, Guangjie Yuan, Gaoyuan Wang, Zonghui Wu, Xingcong Zhao

**Affiliations:** ^1^Education Science College, Guizhou Normal College, Guiyang, China; ^2^College of Electronic and Information Engineering, Southwest University, Chongqing, China; ^3^Chongqing Collaborative Innovation Center for Brain Science, Southwest University, Chongqing, China; ^4^College of Music, Southwest University, Chongqing, China; ^5^Southwest University Hospital, Southwest University, Chongqing, China

**Keywords:** simultaneous EEG-fMRI, DC shifts, EEG, BOLD, auditory

## Abstract

Event-related fMRI have been widely used in locating brain regions which respond to specific tasks. However, activities of brain regions which modulate or indirectly participate in the response to a specific task are not event-related. Event-related fMRI can't locate these regulatory regions, detrimental to the integrity of the result that event-related fMRI revealed. Direct-current EEG shifts (DC shifts) have been found linked to the inner brain activity, a fusion DC shifts-fMRI method may have the ability to reveal a more complete response of the brain. In this study, we used DC shifts-fMRI to verify that even when responding to a very simple task, (1) The response of the brain is more complicated than event-related fMRI generally revealed and (2) DC shifts-fMRI have the ability of revealing brain regions whose responses are not in event-related way. We used a classical and simple paradigm which is often used in auditory cortex tonotopic mapping. Data were recorded from 50 subjects (25 male, 25 female) who were presented with randomly presented pure tone sequences with six different frequencies (200, 400, 800, 1,600, 3,200, 6,400 Hz). Our traditional fMRI results are consistent with previous findings that the activations are concentrated on the auditory cortex. Our DC shifts-fMRI results showed that the cingulate-caudate-thalamus network which underpins sustained attention is positively activated while the dorsal attention network and the right middle frontal gyrus which underpin attention orientation are negatively activated. The regional-specific correlations between DC shifts and brain networks indicate the complexity of the response of the brain even to a simple task and that the DC shifts can effectively reflect these non-event-related inner brain activities.

## Introduction

The entire response of the brain to a specific task is hard to study due to the complexity of the brain's response. Besides the event-related response in the core responding areas, there always be some brain regions whose ongoing activities modulate the response of the core areas (Vuilleumier et al., 2005; Crottaz-Herbette and Menon, 2006; David et al., 2017; O'Craven et al., 2018). Ongoing activities of these regulatory areas do not fluctuate in the event-related way (Sadaghiani et al., 2009; Walz et al., 2014). Traditional functional magnetic resonance imaging (fMRI) utilizes blood-oxygen-level-dependent (BOLD) contrast to locating brain areas whose response complies with the event-related mode (Ogawa et al., 1992; Yuan et al., 2013), whereas localization of these non-event-related activities is beyond its capacity. EEG and fMRI have a notably complement each other (Jorge et al., 2014), and so a combination of EEG and fMRI may have the potential to locate these ongoing activities.

Selecting one meaningful EEG feature is often the first challenge in EEG-fMRI studies. The most often used EEG features are epileptic activity, single trial variability and fast oscillations like alpha and beta activities, etc. (Murta et al., 2015). Activities of EEG are commonly categorized as ultra-slow activity (0~0.1 Hz), delta (0.1–4 Hz), theta (4–8 Hz), alpha (8–12 Hz), beta (12–30 Hz), and gamma (>30 Hz) (Speckmann and Elger, 2004). Due to historical reasons, naming of ultraslow activity of EEG is somewhat confusing. The term DC shifts has been widely used in older literature, while terms like infra-slow oscillations, ultra-slow activities, slow waves, etc. are also often used in recent papers. In order to conform to older literature, we used the term DC shifts to refer to ultra-slow activity (0~0.1 Hz) of EEG. DC shifts are often treated as noise in EEG studies (Palva and Palva, 2012) and the connections between DC shifts and neural activities are often neglected (Northoff, 2017).

However, it has to be noted that DC shifts-fMRI have the potential to analyze inner brain activity. First, activity of cortex induces hemodynamic fluctuations, meanwhile DC shifts can be generated by hemodynamic fluctuations (Vanhatalo et al., 2003; Voipio et al., 2003). Second, DC shifts were found to be regional-specific in simultaneous EEG- magnetoencephalography (MEG) studies (Leistner et al., 2007, 2010; Sander et al., 2007; Mackert et al., 2008). Third, during the resting state, DC shifts were found to reflect the fluctuations of intrinsic networks (Hiltunen et al., 2014). Meanwhile, a task-state study (Haufe et al., 2018) using audio-visual stimuli showed that there are links between fMRI and DC shifts. Furthermore, brain computer interface studies found connections between body movements and DC shifts (Mackert et al., 2004; Banville and Falk, 2016). We reasoned that DC shifts at least can index of part of inner brain neural activity.

Tonotopic mapping of the human auditory cortex has been investigated in great detail using neuroimaging methods (Saenz and Langers, 2014). In these studies, pure tones or their varieties in various frequencies were presented to subjects in random or pseudorandom order (Lauter et al., 1985; Wessinger et al., 1997; Bilecen et al., 1998; Talavage et al., 2004; Humphries et al., 2010; Thomas et al., 2015; Leaver and Rauschecker, 2016). Under this paradigm, auditory cortex, including Heschl's gyrus (HG), planum temporale (PT), part of superior temporal gyrus (STG) and planum polare (PP), was the core area that activated, while areas outside auditory cortex were either hardly activated or neglected by researchers (Bilecen et al., 1998; Bendor and Wang, 2005; Humphries et al., 2010; Langers and van Dijk, 2012; Ahveninen et al., 2016).

It is questionable that only the auditory cortex was activated during the paradigm of tonotopic mapping of auditory cortex. Auditory cortex is not an isolated area but is tightly connected to other cortexes, such as the somatosensory cortex (Budinger and Scheich, 2009; Beer et al., 2011), olfactory cortex (Budinger et al., 2006; Budinger and Scheich, 2009) and visual cortex (Eckert et al., 2008; Beer et al., 2011). A former work (Li et al., 2017) proposed that besides the auditory cortex, portions of parietal and occipital lobes also take part in the late stage of processing of pure tones. In this study, we performed a resting state and a pure tone listening task to study the correlation between DC shifts and BOLD signal during task/resting state. We hypothesis that the correlation of DC shifts and BOLD signal should be different between task/resting state. Besides, contrasting to resting state, a pure tones listening task requires participants to focus and sustain their attention to the incoming tones, and to maintain adequate alertness during the whole study session. Thus, we hypothesize that the sustained attention networks were correlated with DC shifts during the pure tone listening task.

## Materials and Methods

### Participants

Simultaneous EEG-fMRI data were collected from 50 right-handed young and healthy subjects (25 male, 25 female; ages 17–25years). Hearing thresholds of all subjects were tested to make sure that none of the participants had hearing deficits (From 200 to 6,400 Hz, hearing thresholds were better than 20 dB HL). All the subjects were native Chinese speakers. This experiment's protocols were approved by the Institutional Review Board of the Southwest University and all experiments were performed in accordance with relevant guidelines and regulations. Each subject signed the informed consent. For participants under the age of 18 years old, informed consents have been obtained from their parents.

### Stimulus Presentation

**Figure 1:**
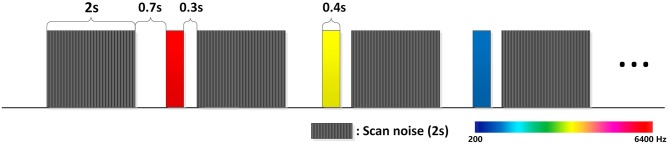
Pure tones are presented during the delay of the EPI scans. This can avoid the acoustic and electromagnetic noise from the fMRI. Shade in this figure represents EPI scans and color represents pure tones. The EPI scan is 2 s and the delay is 1.4 s. Pure tones were presented 0.7 s before the next scan. Stimuli were randomly presented; each kind of stimulus was presented 72 times in total.

We used a classical paradigm which is often used in tonotopic mapping studies of auditory cortex (Nourski et al., 2014; Saenz and Langers, 2014). Pure tones in six frequencies (200, 400, 800, 1,600, 3,200, 6,400 Hz) were created in Matlab. All of them were sine waves and last 0.4 s. If a tone in sinusoidal wave was truncated suddenly there will exist a falling edge and produce a strange sound. So we add a 10-ms exponential rise and fall envelopes to the beginning and ending of all tones. The tones need to be normalized, we used the Bruel and Kjaer 2236 sound meter to test the loudness of the tones and adjust the sound amplitude in matlab and finally all the tones were set to A-weighted 78 dB. Pure tones were randomly presented and in some trials there was no sound presented, these “silent” trials were also randomly presented. To avoid the interference of scan noise, all the tones were presented in the interval between scans (see Figure 1). Experiments were conducted in three runs, and each kind of stimulus including “silent” was repeated 24 times in each run, so each kind of stimulus was repeated 72 times in total. There was a 2–3 min break between runs. During the experiment, subjects were instructed to lie still, keep awake and listen to the tones. No specific task was performed during the experiment.

### FMRI Acquisition and Analysis

FMRI images were acquired at the imaging center of Southwest University using a 3T Siemens scanner. To avoid the interference of scanner noise to pure tones, echoplanar images (EPI) were not continuously acquired but separated by a 1.4 s delay. Pure tones were presented in these delays. By doing this, not only could the scanner noise be avoided, but also the fMRI gradient artifacts to event-related response (ERPs) could be eliminated (Humphries et al., 2010). The repetition time (TR) was 3.4 s,and taking into account the 1.4 s delay time, the EPIs acquired time was 2 s. Echo time was set as 26 ms. Voxels were in 3^*^3^*^3 mm scales, 32 slices in total, flip angle was 90. After the fMRI scans, a high resolution anatomical T1 scan was performed. T1 images were scanned in 1^*^1^*^1 scales, 176 slices in total. TR was set as 1900 ms, TE was set as 2.52 ms.

Traditional fMRI preprocess and analysis were performed in surface space using FreeSurfer (http://surfer.nmr.mgh.harvard.edu). T1 images were used to reconstructed cortical surface. All the 50 subjects' cortical surfaces were used to construct an average surface to perform the statistical analyses and display the results. The preprocess steps for fMRI data include head motion correction, slice timing correction, skull stripping. Then fMRI data were spatial normalized to the average surface which was constructed by former steps. After that, a 5 mm FWHM kernel was used to smooth the fMRI data.

For each subject, the onset times of six kinds of stimuli and ‘silent' trials were convolved with a Haemodynamic response function (HRF) offered by FreeSurfer and entered into a GLM to perform the first level analysis. Then the second level analysis (random effects group analysis) was carried out based on the first level results. A surface-based correction for multiple comparisons was executed with cluster-based threshold. The data were initially thresholded at *p* <0.05 and then corrected at an alpha level of 0.05.

The response frequency of a point was calculated with the follow rules. If only one frequency was significant in a vertex, then the vertex was marked with this frequency. But if more than one frequencies were significant in a vertex, the weighted mean value of these frequencies was calculated and was set as the response frequency of the vertex, that is$\bar{f}=\sum_{1:\;n}T_{i}f_{i}{/}_{\underset{1\;:\;n}{\sum^{\text{​}}}T_{i}}$, n is the number of frequencies that are significant in the vertex.

### EEG Acquisition and Analysis

We used a 32 electrode non-magnetic MRI-compatible EEG system (BrainAmp MR plus, Brain products, Munich, Germany) to record the EEG data under simultaneous fMRI scans. According to the Nyquist Theorem, the sampling rate must be more than twice the maximum frequency component of the signal being measured. The frequency range of fMRI noise could reach 2 kHz (Allen et al., 2000), so the sample rate of EEG was set at 5 kHz to record the fMRI noise and then eliminated it. Before the scan, impedances of all electrodes were tested and all of the values were kept below 10 kΩ. The locations where the electrodes were placed was in accordance with the international 10/20 system. An electrocardiogram (ECG) electrode was attached to the back of subjects near the position of heart to detect the ECG. Signals were digitized at a passband of 0–250 Hz.

The EEG was processed offline using eeglab13 (Delorme and Makeig, 2004) in matlab (Mathworks, Natick, MA) and Brain Vision Analyzer (version 2.1, Brain Products). In Analyzer 2.1, EEG data was re-referenced to average TP9/TP10. FMRI gradient artifacts were removed using a local average artefact procedure (Allen et al., 2000) and a cutoff frequency of 70 Hz was selected. Then EEG was down-sampled to 500 Hz. To remove the ballistocardiogram (BCG) artifacts, EEG data was exported to eeglab13. The toolbox FMRIB 2.0 (Niazy et al., 2005) was used to detect and remove the BCG-related artifacts. The parameter “number of PCs to use” was set as 7 (if less than 3, the signal-noise ratio of ERP would be low). After BCG artifacts removal, EEG was imported into Analyzer 2.1 again, to remove the ocular artifact using “Ocular remove using ICA.” After that, EEG data was exported to eeglab13 for further analysis. Traditional ERPs were extracted for six kinds of stimuli. In this study, we analysed ERPs from a pooled electrode comprised of FC1, FC2, and Cz, all located in a frontocentral area, an area whichhas been proven to be the area of greatest N1 amplitude (Näätänen et al., 1988; Herrmann et al., 2013b). Epochs were extracted from −200 to 600 ms from the onset of the pure tones. Baseline was set to the −200~0 ms. The correlation between N1 amplitude and frequencies was calculated (see **Figure 3**). EEG was then low passed (<0.1 Hz) to obtain the DC shifts of EEG and then entered the joint EEG-fMRI analysis.

### Joint DC Shifts-fMRI Analysis

**Figure 2:**
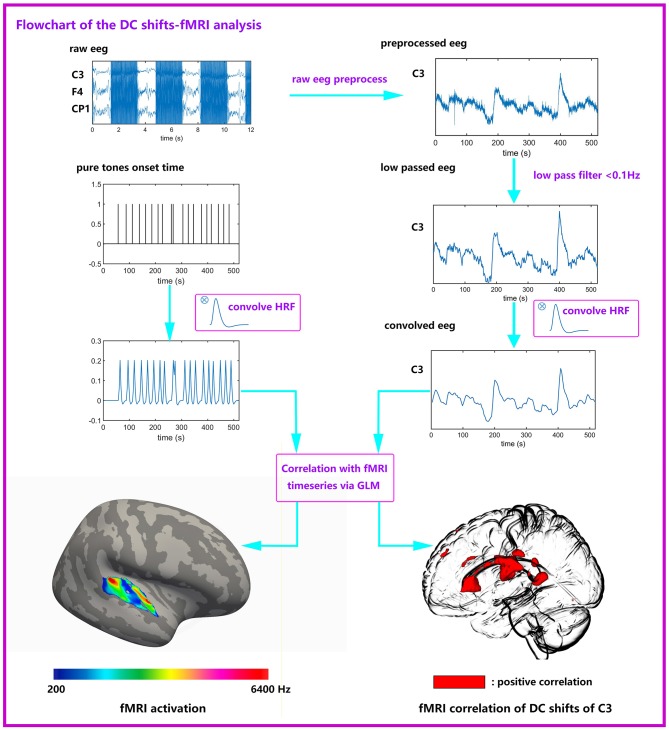
Flowchart of the joint DC shifts-fMRI analysis. Raw EEG data were preprocessed (gradient artifacts remove, BCG artifacts remove, ocular artifacts remove), and low passed (<0.1 Hz). Then EEG data convolves with HRF and entered a GLM together with six kinds of stimuli. The EEG data comes from one subject, it is the first 520 s of the total data. It can be seen that the DC shifts are obvious. The pure tones onset time is an example, it is not from the true data.

The flowchart of joint DC shifts-fMRI analysis is shown in Figure 2. In the joint DC shifts-fMRI analysis, the fMRI data for each subject were analyzed in the volumetric space mni305 using Matlab (Mathworks Inc, Natick, MA). Data were pooled across runs. A standard preprocessing processing stream was conducted to fMRI data under the guidance of the SPM12 Manual (http://www.fil.ion.ucl.ac.uk/spm/). The steps included head motion correction, slice timing correction, coregistration to anatomic structure, spatial normalization and spatial smoothing. Preprocessed fMRI data then entered the joint analysis with EEG DC shifts. A general linear model (GLM) was applied to each voxel time series.

Two kinds of regressors were made. One kind of regressors were designed for conventional fMRI analysis, formed by convolving the onset times of each kind of pure tones and a canonical hemodynamic response function. Another kind of regressors were designed for the DC shifts-fMRI analysis, which were extracted from each electrode's DC shifts amplitude. The extracting time point was set at the beginning of each EPI. These regressors were convolved with a canonical hemodynamic response function to enter GLM for DC shifts-fMRI analysis. Both these two kinds of regressors entered into single-subject fixed-effects regression analyses; on the group level, random effects analyses were performed using one-way ANOVA. Activations to targets are significant at *P* <0.05, cluster-extent family-wise-error (FWE) corrected (voxel-height threshold *P* <0.005, extent 10 voxel). This analysis method was adapted from Eichele et al. (2005).

### Correlation Between Time-Shifted DC Signal and BOLD Signal

To get a more detailed picture of the relationship between the DC signal and BOLD signal, we selected six seed points according to the results of 2.5 Joint DC shifts-fMRI analysis. We forward shifted the DC signal with time shift from 0 to 6 s, 0.2 s each space, and calculated the correlation between the DC signal and BOLD signal in those six seed points with GLM. It's worth noting that in this analysis, the time-shifted DC signal was not convolved with HRF, but rather they directly entered GLM together with traditional regressors. Besides this, the other steps are all the same with 2.5. The names, locations and MNI coordinates of these six seed points were A: Caudate_R (20, −6, 26), B: Thalamus (−12, −8, 14), C: Caudate_L (−14, 24, 4), D: Frontal_Mid_R (50, 12, 4), E: Precuneus_R (18, −74, 52), F: Precuneus_L (−22, −82, 38). Each seed point concluded with 3 ×3 ×3 = 27 voxels and the correlation result was the mean value of the T value of these 27 voxels.

## Results

To prove DC shifts-fMRI can reveal a more complete response of the brain to stimuli, we acquired simultaneous recorded fbEEG-fMRI from 50 young and healthy subjects during a classical paradigm of tonotopic mapping of auditory cortex. Plenty of fMRI studies have showed that the response of the brain to this paradigm is concentrated on auditory cortex (see Introduction) while our DC shifts-fMRI results revealed that there are other activations that can't be neglected. Traditional fMRI and ERP were also analyzed to compare with previous findings.

### FMRI Results

**Figure 3:**
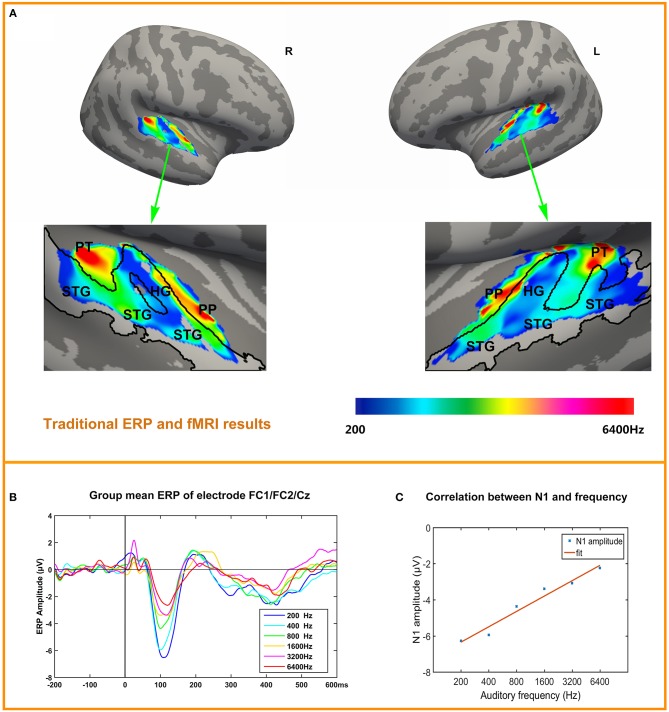
ERP and tonotopy maps. **(A)** Bilateral hemisphere tonotopy map for the group mean. Colored regions are areas that showed significant differences across the six conditions (*p* <0.05, corrected). Regions are color-coded according to the frequency responded. STG, Superior Temporal Gyrus; HG, Heschl's Gyrus; PP, Planum polare; PT, Planum Temporale. **(B)** ERP to six conditions in pooled electrode FC1/FC2/Cz, N1 is obvious and the amplitude of N1 decreases with frequencies increase. **(C)** Correlation of N1 amplitude and frequency.

Tonotopy maps were displayed in Figure 3A. The bilateral tonotopy maps were similar. It could be seen that the Heschl's gyrus (HG) divided the high frequencies response areas into two parts. HG mainly responded to the 200 Hz (blue), while another area responding to 200 Hz was at the lateral posterior STG. Two high frequencies response areas (red) were located anterior and posterior to the HG, in planum polare and planum temporale. Intermediate-frequency areas were located between low and high areas.

### Average ERPs

Figure 3B depicts the time courses of the averaged response to each tone, in each condition in a fronto-central electrode cluster FC1/FC2/Cz showed the strongest responses in the ERP. The most obvious components are P1 (10~50 ms), N1 (100~120 ms), P2 (200~240 ms), N4 (400~450 ms). Figure 3C depicts the correlation between N1 amplitude (pooled electrode FC1/FC2/Cz) and frequency (log-scaled). Correlation is significant (*r* = 0.9815, *p* = 0.0005). Correlation between P1, P2, N4 and frequency (log-scaled) are not significant, with P1 (*r* = 0.0544, *p* = 0.9185), P2 (*r* = −0.8194, *p* = 0.046), N4 (*r* = 0.5490, *p* = 0.2592). The individual's N1 amplitude was showed in the Supplementary Material.

### DC Shifts–fMRI Results

**Figure 4:**
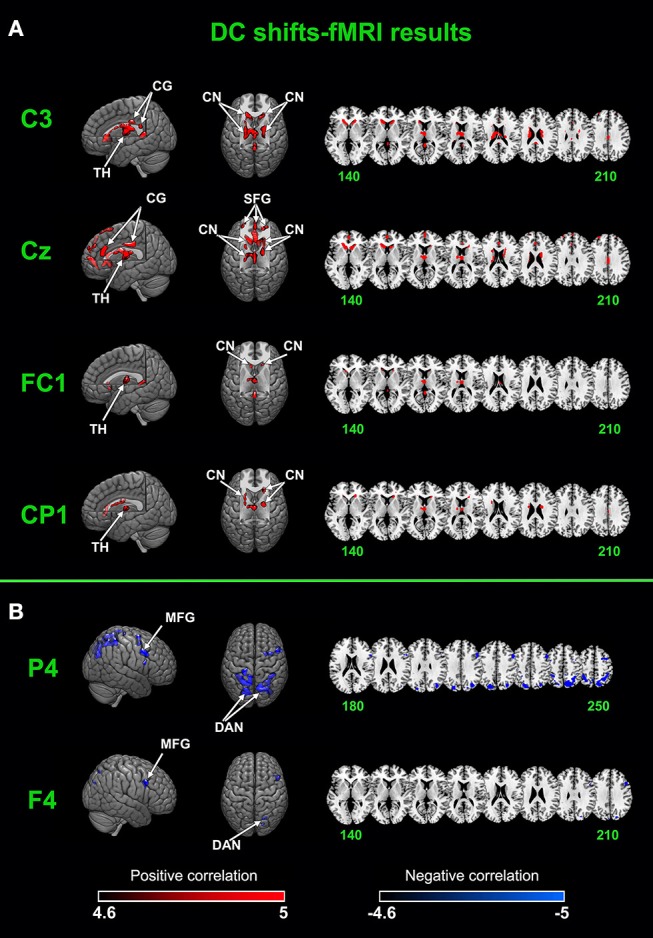
Joint DC shifts-fMRI results. **(A)** Brain networks that positively correlated with DC shifts in C3, CZ, FC1, and CP1. **(B)** Brain networks that negatively correlated with DC shifts in F4 and P4. CG, cingulate gyrus; TH, thalamus; CN, caudate nucleus; SFG, superior frontal gyrus; MFG, middle frontal gyrus; DAN, dorsal attention network.

After removing fMRI and physiological artifacts from raw EEG data, we low-pass filtered (<0.1 Hz) the EEG to acquired DC shifts. DC shifts were down-sampled to the same frequency of FMRI of 1/3.4 Hz. Sampling times were set at the beginning of each EPI. The down-sampled DC shifts were convolved with HRF and entered the GLM analysis with fMRI time series. Two results were shown in Figure 4 and Table 1.

**Table 1:** DC shifts-fMRI analysis results.

					**MNI coordinates**		
**Electrodes**	**Region**	**Cluster size**	**T**	**±**	**x**	**y**	**z**
C3	Thalamus	590	6.43	pos	−12	−8	14
	Caudate_L	246	5.49	pos	−14	24	4
	Caudate_R	159	5.43	pos	18	26	2
	Caudate_R	173	5.50	pos	20	−6	26
	Corpus callosum	111	6.01	pos	0	−46	10
	Posterior cingulate	40	5.19	pos	2	−38	22
	Anterior cingulate	21	4.76	pos	0	28	28
	Middle cingulate	52	5.05	pos	2	−20	34
Cz	Caudate_R	397	5.96	pos	8	20	−2
	Caudate_L	246	6.27	pos	−10	20	0
	Thalamus	284	6.70	pos	−2	−8	10
	Anterior cingulate	175	5.66	pos	0	52	2
	Anterior cingulate	115	5.33	pos	0	32	24
	Superior frontal cortex	58	5.22	pos	−26	52	34
	Superior frontal cortex	107	5.38	pos	18	44	46
	Superior frontal cortex	142	6.01	pos	−4	36	60
	Middle cingulate	135	5.46	pos	2	−20	34
FC1	Thalamus	178	6.46	pos	−2	−10	12
	Posterior cingulate	72	5.28	pos	2	−44	10
	Caudate_L	24	5.12	pos	−8	20	−2
	Caudate_R	25	4.89	pos	18	24	6
CP1	Thalamus	135	6.60	pos	−2	−10	10
	Caudate_R	125	5.74	pos	20	30	−2
	Caudate_R	65	5.26	pos	20	−6	26
	Caudate_L	53	5.20	pos	−20	8	20
	Superior frontal gyrus	16	5.05	pos	−2	36	60
F4	Middle frontal gyrus	114	5.44	neg	48	16	36
	Precuneus_R	26	4.92	neg	20	−82	32
	Parietal_sup_R	31	5.3	neg	22	−74	54
P4	Precuneus_L	127	5.45	neg	−22	−82	38
	Precuneus_R	118	6.45	neg	34	−76	36
	Frontal_mid_R	98	5.96	neg	50	12	40
	Precuneus_R	193	6.46	neg	18	−74	52
	Parietal_sup_L	164	5.55	neg	−30	−52	56
	Parietal_sup_R	54	5.40	neg	26	−56	56
	Postcentral_R	76	5.54	neg	38	−44	62
	Precuneus_L	94	5.75	neg	−8	−48	64
	Postcentral_L	53	5.81	neg	−22	−38	70

A cingulate-caudate-thalamus network consisting of the cingulate gyrus, the caudate nucleus, the thalamus and the superior frontal gyrus was found to be positively correlated with DC shifts in electrodes of C3, Cz, FC1, CP1. In C3, bilateral caudate nucleus, the cingulate gyrus and the thalamus were activated. In Cz, the superior frontal gyrus, the cingulate gyrus, bilateral caudate nucleus and the thalamus were activated. In FC1 and CP1, the activated regions are lesser, with bilateral caudate nucleus and thalamus mainly activated.

The dorsal attention network was found to be negatively correlated with DC shifts in P4 and F4. The middle frontal gyrus was also found negatively activated in these two electrodes' results. It's worth noting that all these results passed FWE (*p* <0.05) correction.

### Correlation Between Time-Shifted DC Signal and BOLD Signal

**Figure 5:**
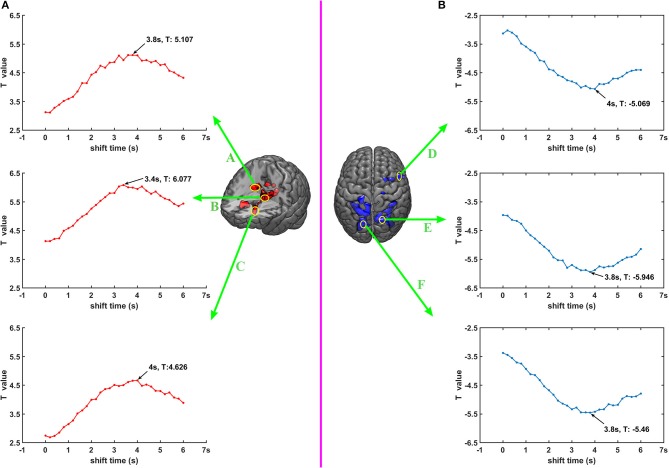
**(A,B)** Correlation between time shifted DC and BOLD.

The second level analysis results between time-shifted DC signal and BOLD signal in seed points are shown in Figure 5, with Figure 5A showing the results of seed points A, B and C, with their BOLD signals correlated with DC signal of electrode C3. Figure 5B shows the results of seed points D, E and F, and their BOLD signals are correlated with DC signal of electrode P4. It can be seen that for both these six seed points, the correlation between time-shifted DC signal and BOLD signal peaked when the time shift was about 3.4~4 s.

## Discussion

Generally, the auditory cortex is thought to be the dominantly activated area under the paradigm of tonotopic mapping in the auditory cortex and the activation of other areas is negligible. In the current study, we used DC shifts-fMRI in combination with traditional fMRI and ERP analyses to investigate a more complete response of the brain during this paradigm. Participants were asked to listen to the tones and no other task was performed. Our traditional fMRI results showed that the auditory cortex was the only area that activated during the paradigm and the tonotopy organizations were in line with early findings of other researchers (Humphries et al., 2010; Saenz and Langers, 2014). Our DC shifts-fMRI results showed that BOLD signals in a cingulate-caudate-thalamus network were positively correlated with scalp recorded DC shifts in electrode of C3, CZ, FC1, CP1. While BOLD signals in the dorsal attention network and the right middle frontal gyrus were negatively correlated with DC shifts in F4 and P4. The cingulate-caudate-thalamus network is the neural substrate of sustained attention (Petersen, 1990; Pardo et al., 1991; Sarter et al., 2001; Oken et al., 2006; Sadaghiani et al., 2010b) while the dorsal attention network and the right middle frontal gyrus are related to attention orientation (Corbetta et al., 2008; Japee et al., 2015). Resting state data was acquired before the paradigm, but both traditional fMRI and DC shifts-fMRI showed that there was no activation during resting state.

In line with previous findings, the conventional fMRI analyses showed pronounced activation in the auditory cortex. The tonotopy organization of our work is consistent with the typical tonotopic patterns (Lauter et al., 1985; Pantev et al., 1988; Bilecen et al., 1998; Talavage et al., 2004; Humphries et al., 2010; Saenz and Langers, 2014). The low frequencies areas are mainly located at HG, flanked by two high-frequency zones posteromedially toward the planum temporale and anteromedially toward the circular sulcus. The ERP results are also consistent with previous findings. The N1 component has been shown to be modulated as a function of sound frequency (Picton et al., 1978; Näätänen et al., 1988; May et al., 1999; Herrmann et al., 2013a,b), and our ERP results nicely converge with these findings.

### Mechanisms Linking DC Shifts and fMRI

The DC shifts may come from two mechanisms: the neuronal and non-neuronal mechanisms, both of which link DC shifts to BOLD signals. At early times, DC shifts were thought to be generated by neuronal mechanisms. Basing on epileptic activity in animal experiments, (Birbaumer et al., 1990; Roland, 2002; Speckmann and Elger, 2004) research supported the opinion that DC shifts are generated by tonic depolarization of the apical dendrites of cortical pyramidal neurons. Besides somatodendritic neuronal dipoles, the epileptic activity of glial cells (Caspers et al., 1987; Laming et al., 2000) and extracellular potassium concentration (Staschen et al., 1987; Voipio and Kaila, 2000) are also involved in the generation of DC shifts. All of the above can be simplified as neuronal mechanisms of the generation of DC shifts.

However, currently, the more prevailing hypothesis regarding the mechanisms of DC shifts generation is the non-neuronal mechanisms, to be specific, it comes from the fluctuations of potential difference across blood brain barrier. By directly manipulating intracranial hemodynamics, Vanhatalo et al. (2003) found that hemodynamic changes could arouse DC shifts and these shifts are apparently not related to neuronal activities. They deduced that these DC shifts are produced by changes in potential difference across blood brain barrier. (Voipio et al., 2003) found that hypo- and hypercapnia can elicit DC shifts and they thought that these DC shifts are not from a neuronal origin but from the potential difference across the blood-brain barrier that can be recorded on the scalp. Recent DC shifts-fMRI studies support the view that scalp recorded DC shifts stem from blood brain barrier (Palva and Palva, 2012; Hiltunen et al., 2014). Blood brain barriers linked DC shifts and hemodynamic fluctuations, vesting the ability of DC shifts in revealing inner brain activities.

### Attention Networks Fluctuate During the Pure Tones Listening Task

In addition to our fMRI and ERP results, which are highly consistent with previous findings, the DC shifts-fMRI revealed that the cingulate-caudate-thalamus network which underpins sustained attention and the dorsal attention network and the right middle frontal gyrus which underpin attention orientation, were also activated during the paradigm. Sustained attention, tonic alertness and vigilance are three terms with the same meaning, and psychologists use these terms to describe an ability to sustain attention to a task for a period of time (Oken et al., 2006). Sustaining attention to the task at hand, such as following a lecture at school or maintaining focus while driving, is a crucial part of everyday life (Jangraw et al., 2018). It has been claimed that maintaining attention and engagement on a relatively monotonous task over time requires sustained attention (Unsworth et al., 2018). The pure tones listening task is a typical scenario that need sustained attention.

The cingulate-caudate-thalamus network, including the anterior, middle, and posterior cingulate gyrus, bilateral caudate nucleus, the thalamus, and the superior frontal cortex, was found to be positively activated during the tonotopic mapping paradigm. Previous studies revealed that this network underpins the maintenance of sustained attention. Despite the subtle differences, this network is consistent with the intrinsic connection network proposed by (Dosenbach et al., 2007; Seeley et al., 2007). They proposed that this network controls goal-directed behavior through the stable maintenance of task sets. Coste and Kleinschmidt (2016) found a cingulate-opercular network whose higher pre-stimulus activity results in a faster response speed to targets and thus underpins the deployment of sustained attention. This is a cingulate-opercular overlap with the cingulate-caudate-thalamus network in the thalamus and the cingulate cortex. In a study of detecting auditory near-threshold stimuli, (Sadaghiani et al., 2009) found that the higher prestimulus activity of a cingulate-insular-thalamus network facilitates the performance of perception. In the later paper of Sadaghiani et al. (2010b), they proposed an interpretation that the cingulate-insular-thalamus network underpins the maintenance of sustained attention. In an auditory alertness study (Sturm et al., 2004), a network including cingulate, thalamus and inferior parietal structures was found subserving sustained attention. Similar to our results, Sturm et al. (1999) found a frontal-parietal-thalamus network activated during a visual intrinsic alertness study. Hinterberger and colleagues used simultaneous EEG-fMRI recordings to uncover the relevant areas of brain activation during self-regulation. Event-related slow waves were successfully related to the BOLD response in the caudate nucleus and thalamus (Hinterberger et al., 2003, 2005). The structure of the cingulate-caudate-thalamus network is appropriate for underpinning sustained attention. The thalamus connects closely with the whole cerebral cortex, suited for maintenance of alertness (Scheibel and Scheibel, 1967). The caudate nucleus constitutes a part of the sustained attention network (Sadaghiani et al., 2010b). It is structurally tightly connected with the thalamus (Alexander, 1986) and plays an important role in transporting information between the thalamus and the prefrontal cortex (Rothwell, 2011). The cingulate cortex is the major cognitive control region, playing roles in adaptive top-down control (MacDonald et al., 2000; Kerns et al., 2004). Above all, it could be concluded that the cingulate-caudate-thalamus network functionally and structurally underpins the maintenance of sustained attention.

The DC shifts-fMRI results also showed that during the tonotopic mapping paradigm, the dorsal attention network is negatively correlated with DC shifts in P4 and F4. The dorsal attention network consists of intraparietal sulcus and the junction of the precentral and superior frontal sulcus (Fox et al., 2006). This network is generally believed to be involved in top-down orienting of attention (Corbetta and Shulman, 2002; Fox et al., 2006; Proskovec et al., 2018; Zhou et al., 2018). The activity of this network increases when there is a cue indicating when, where, or to what participants should pay their attention (Giesbrecht et al., 2003; Corbetta et al., 2005; Fox et al., 2006). Other than the role in attention orientation, the dorsal attention network was also thought to anti-correlated with the sustained attention. In a resting state experiment, the dorsal attention network was found negatively correlated with the alpha global field power which is the most consistent reported EEG-hallmark of sustained attention (Sadaghiani et al., 2010b). In the near-threshold stimuli task, (Sadaghiani et al., 2009) found the higher prestimulus activity of the dorsal attention network disrupted the perception performance. They explained that the dorsal attention network supported the processing of attention orientation and this function might compete with processing of sustained attention. Besides the negative activation of dorsal attention network, the right middle frontal gyrus was also negatively activated during the tonotopic mapping paradigm. Similar to the function of the dorsal attention network, the right middle frontal gyrus has also been found playing a role in attention orientation (Corbetta et al., 2008; Japee et al., 2015). Taken together, our results support the view of (Sadaghiani et al., 2009) that there is competitive relationship between attention orientation networks and sustained attention networks.

### Reasons why Attention Networks Fluctuate During the Pure Tones Listening Task

In the paper of (Hiltunen et al., 2014), DC shifts were discussed in two categories: event-related DC shifts and spontaneous DC shifts. Event-related DC shifts were found in situations of sensory stimuli (Walter et al., 1964), motor actions (Kornhuber and Deecke, 1965), cues preceding to-be-attended-to stimuli (Gonzalez-Rosa et al., 2011; Werner et al., 2011; Zanto et al., 2011), long-term memory (Khader et al., 2007; Kizilirmak et al., 2012), etc. Spontaneous DC shifts refer to DC shifts present during resting state. Both event-related and spontaneous DC shifts were found correlated with BOLD signals (Leistner et al., 2007, 2010; He and Raichle, 2018).

However, it has to be noted that the DC shifts presented during the pure tone listening task are neither event-related nor spontaneous. The traditional event-related fMRI doesn't have the ability to locate these two sustained attention networks, indicating that the activities of these two networks and the DC shifts induced by them are not event-related. In a resting state, these two networks were not found by DC shifts-fMRI, indicating that the activities of these two networks are not spontaneous. Each system of the brain is not an independent section. Perception process of a system, e.g., auditory system, is not isolated but correlates with ongoing activity fluctuations of other cerebral cortexes (Sadaghiani et al., 2010a,b). There are some ongoing activities of the cerebral cortex's impact the information process while the ongoing activities themselves contain no specific information (Oken et al., 2006). It could be inferred that the activities of these attention networks and the DC shifts induced by them modulate the response of the auditory cortex.

The participants were asked to listen to the pure tones played, with the constant tones during the paradigm prompting the subjects maintain sustained attention. It is reasonable that the sustained attention network is activated during the task. The state of readiness to respond to stimuli is termed as ‘sustained attention level', the sustained attention level is not static over time (Sarter et al., 2001). The tonotopoic mapping paradigm lasts nearly 40 min, and although the stimuli rarely change, the sustained attention level inevitably fluctuates over time. The fluctuations of the attention networks induce the fluctuations of hemodynamics, and hence induce the fluctuations of potential differences across the blood brain barrier. These potential difference fluctuations spread through the skull and are recorded by the fbEEG. There is competitive relationship between sustained attention and orientation of attention, which can explain why during the task the neural substrates underpin these two functions fluctuate inversely. With DC shifts offering the index of inner brain ongoing activity and fMRI offering the location, DC shifts-fMRI hence has the ability to localize ongoing activity which couldn't be localized by traditional event-related fMRI.

This pure tone listening process is not the only cognitive process that is found accompanied by slow oscillations, slow oscillations were also found in some other cognitive processes (Kucyi et al., 2018). Using a unique continuous performance task, Kucyi et al. (2016) found that the intense mind-wandering co-occurred with high-amplitude slow oscillations of the default mode network. In some behavioral fluctuation studies, spontaneous fluctuations in response-time variability have been found correlated with slow oscillations of the default network, dorsal and ventral attention networks (Esterman et al., 2012; Rosenberg et al., 2015; Kucyi, 2018). Moreover, many perception fluctuation studies found that perception fluctuation is correlated with slow ongoing activity in large-scale higher order brain networks (Boly et al., 2007; Coste et al., 2011; Sadaghiani et al., 2015).

It's worth noting that when DC shifts were moved forward around 3.8 s, the *T*-value would reach the max value in selected seed points. This phenomenon suggests that when listening to pure tones, DC shifts are leading BOLD signal about 3.8 s. The delay of BOLD signals may stem from the slow signature of BOLD (Jorge et al., 2014), and that neuronal activity needs a complicated process and several seconds to cause fluctuation of BOLD (Smith et al., 2001). This finding, to some extent, is in line with the resting state study of Grooms et al. (2017). They found that in a resting state, some brain networks' BOLD signal was correlated with some electrodes' DC shifts, and part of these correlations reached the peak when the time shift was around 4 s. Besides, some invasive studies investigating correlation between LFP and BOLD proposed that a moved forwards (~4 s) DC signal is correlated with BOLD (Pan et al., 2013; Thompson et al., 2014; Li et al., 2015), and our results are strongly in accordance with their findings.

## Conclusions

In contrast to other EEG features, the DC shifts-fMRI have been rarely studied. To our knowledge, until now DC shifts-fMRI has not been used in studies involving tasks. Using DC shifts-fMRI, we found that the brain responds to a simple pure tones listening task in a more complicated way than previously thought. Networks related to sustained attention and attention orientation were also activated besides the traditional activation in the auditory cortex during the task. Our results show that the traditional fMRI has limitation and DC shifts-fMRI can be a meaningful supplement in locating continuously fluctuating networks.

## Data Availability

The datasets for this manuscript are not publicly available because the datasets for this manuscript are not publicly available because Due to the potential value that has not been fully explored by the current manuscript, survey respondents were assured raw data would remain confidential and would not be shared. Requests to access the datasets should be directed to 648047815@qq.com.

## Ethics Statement

This study was carried out in accordance with the recommendations of APA ethical standards of the ethics committee of Southwest University with written informed consent from all subjects. All subjects gave written informed consent in accordance with the Declaration of Helsinki. The protocol was approved by the ethics committee of Southwest University.

## Author Contributions

QL designed the experiment, analyzed the data and wrote the paper. GY did something in the experiment. GL designed the experiment. GW, ZW, and XZ took part in the experiment and revised the paper.

### Conflict of Interest Statement

The authors declare that the research was conducted in the absence of any commercial or financial relationships that could be construed as a potential conflict of interest.

## Supplementary Material

Figure S1 Individual's N1 amplitude.
