# Supplementary figures and images for: DC Shifts-fMRI: A Supplement to Event-Related fMRI
Source: Front Comput Neurosci. 2019 Jun 12;13:37. doi: 10.3389/fncom.2019.00037 (PMC6581730; doi:10.3389/fncom.2019.00037)

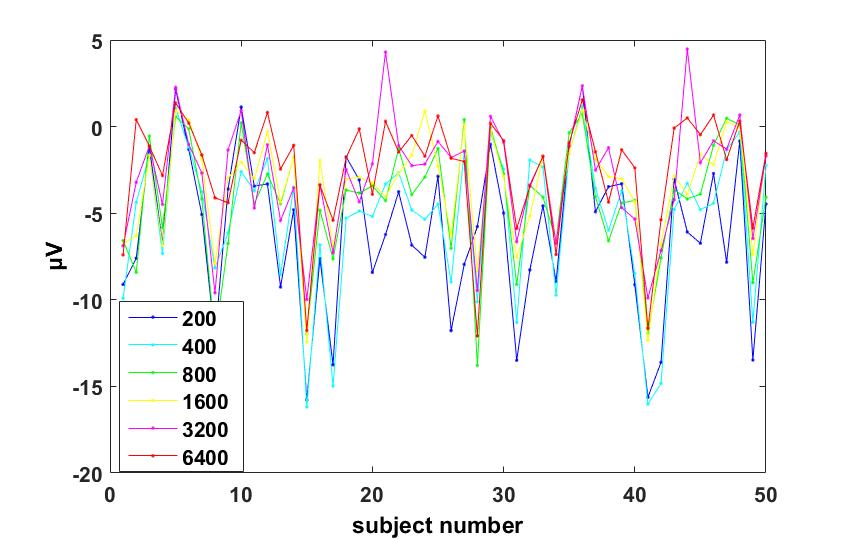

Supplement: Figure S1 — Individual's N1 amplitude. [file Image_1.JPEG]
